# H_2_S Pretreatment Is Promigratory and Decreases Ischemia/Reperfusion Injury in Human Microvascular Endothelial Cells

**DOI:** 10.1155/2021/8886666

**Published:** 2021-04-15

**Authors:** Elisa Zicola, Elisa Arrigo, Daniele Mancardi

**Affiliations:** Department of Clinical and Biological Sciences, University of Turin, Italy

## Abstract

Endothelial cell injury and vascular function strongly correlate with cardiac function following ischemia/reperfusion injury. Several studies indicate that endothelial cells are more sensitive to ischemia/reperfusion compared to cardiomyocytes and are critical mediators of cardiac ischemia/reperfusion injury. H_2_S is involved in the regulation of cardiovascular system homeostasis and can act as a cytoprotectant during ischemia/reperfusion. Activation of ERK1/2 in endothelial cells after H_2_S stimulation exerts an enhancement of angiogenesis while its inhibition significantly decreases H_2_S cardioprotective effects. In this work, we investigated how H_2_S pretreatment for 24 hours prevents the ischemia/reperfusion injury and promotes angiogenesis on microvascular endothelial cells following an ischemia/reperfusion protocol in vitro, using a hypoxic chamber and ischemic buffer to simulate the ischemic event. H_2_S preconditioning positively affected cell viability and significantly increased endothelial cell migration when treated with 1 *μ*M H_2_S. Furthermore, mitochondrial function was preserved when cells were preconditioned. Since ERK1/2 phosphorylation was extremely enhanced in ischemia/reperfusion condition, we inhibited ERK both directly and indirectly to verify how H_2_S triggers this pathway in endothelial cells. Taken together, our data suggest that H_2_S treatment 24 hours before the ischemic insult protects endothelial cells from ischemia/reperfusion injury and eventually decreases myocardial injury.

## 1. Introduction

Cardiovascular diseases represent the leading cause of death and disabilities in the industrialized countries, and ischemic heart disease (IHD) majorly represents them. IHD causes 46% of cardiovascular deaths in men and 38% in women worldwide [1]. One of the major challenges in treating the ischemic heart is to avoid ischemia reperfusion injury (IRI); the limitation of which can result of pivotal importance in case of programmed ischemia/hypoxia such as open-heart surgery, transplantation, or primary percutaneous coronary intervention [2–4].

IRI is the trigger leading to cell death by apoptosis and necrosis, and the causes of the activation of these phenomena are mainly oxidative stress, inflammation, and intracellular Ca^2+^ overload [5, 6]. In addition, reperfusion can also lead to severe ventricular arrhythmia and, as a consequence, death [7]. Low cardiac output, perioperative myocardial infarction, and arrhythmias can, in turn, be the causes of I/R injury following cardiac surgery [8].

Paradoxically, the restoration of blood flow in the ischemic tissue can exacerbate the damage more than the ischemic event itself, causing a phenomenon known as reperfusion injury [2, 9]. Indeed, the restoration of coronary blood flow, although necessary after the ischemic episode, leads to the death of cardiac myocytes that were potentially viable at the onset of reperfusion [10].

Since 1980, many important contributions shed light on the importance of the endothelium in the cardiovascular system, redirecting the investigating approach to the cardiovascular system [5, 6, 11–16]. In the last 40 years, many advances have been made toward a deeper comprehension of the role of different cell types participating in the ischemic *scenario* and the attention on ECs proportionally increased.

In support to these findings, several studies indicate that endothelial cells are as sensitive to ischemia/reperfusion as cardiomyocytes and therefore are critical mediators of cardiac IRI [17–22].

Furthermore, reoxygenation of the ischemic tissue causes oxidative stress: during reperfusion, xanthine oxidase, NADPH oxidase, and the mitochondrial electron transport chain (mETC) generate reactive oxygen species (ROS), mediating an increased myocardial injury [23, 24]. The abovementioned enzymes also provide the greatest amount of superoxide anion in ECs [6].

During IRI, there is a deficiency of vasodilator molecules such as endothelin [25], angiotensin, prostacyclin, and nitric oxide, which directly influence metabolic and contractile function in the adult heart [22, 26]. After the IR insult, ECs undergo changes in cytoskeletal architecture and expression of adhesion molecules (e.g., CAMs and E- and P-selectins). The activation of quiescent ECs induces the recruitment of neutrophils which initiate the inflammatory response. In parallel, ECs permeability increase resulting in the loss of barrier function and capillary leakage [3, 5, 27].

The process of angiogenesis, which is *de novo* formation of micro vessels, in pathological conditions is not only essential to prevent heart failure in the long term but also has the potential to support the recovery of the ischemic myocardium in the days following myocardial infarction. Impairment of myocardial angiogenesis causes a reduction in myocardial perfusion and fatal ischemic cardiomyopathy [28–30]. Moreover, EC injury and vascular function strongly correlate with the cardiac performance following IRI [31].

To prevent pathological remodeling in the structure of blood vessels, ECs produce and release two endogenous gasotransmitters, hydrogen sulfide (H_2_S) and nitric oxide (NO). In particular, H_2_S has been known for many years for being a toxic agent. Nowadays, it is recognized as being a key factor in the regulation of inflammatory and immune response, cardiovascular and nervous systems, and gastrointestinal tract function [32–35]. The cardiac tissue is capable of producing H_2_S endogenously, due to the presence of a discreet quantity of cystathionine-*γ*-lyase (CSE), one of the enzymes responsible for the production of this gasotransmitter [36]. H_2_S can act as a cytoprotectant against oxidative stress and as an antiapoptotic agent by preserving mitochondrial function during ischemia/reperfusion [37, 38].

The identification of the reperfusion injury salvage kinase (RISK) pathway led the way to understand how to improve the clinical outcomes of acute myocardial infarction by mediating a programmed cell survival [9, 39, 40] when activated specifically at the time of reperfusion [41]. The RISK pathway is linked to two signaling cascades: PI3K-Akt and MEK1-ERK1/2. Hu et al. observed not only that preconditioning rat myocytes with NaHS (1–100 *μ*M) increased cell viability but also that if ERK1/2 or Akt were blocked during preconditioning or ischemia there was a significant decrease of the H_2_S cardioprotective effect [42].

Studies on isolated rat liver mitochondria showed a biphasic effect of H_2_S on mETC. Concentrations ranging from 100 nM to 1 *μ*M stimulated electron transport, whereas 10 *μ*M or higher provoked its inhibition [43]. The same bimodal effect has been shown by Pupo et al., where H_2_S induced no effect at the lowest and highest concentrations (0.5 and 100 *μ*M) and a significant cellular response at 1 *μ*M in terms of cell migration and proliferation [44].

Both PI3K-Akt and MEK-ERK1/2 pathways have been demonstrated to be downstream effectors of H_2_S signaling cascade also in ECs, thus promoting migration, proliferation, and angiogenesis [35, 45–48].

Despite the growing literature in the field, it is still not clear whether H_2_S could be more effective as a preconditioning or postconditioning agent [7, 19, 40, 42, 49, 50].

In our study, we decided to investigate the role of H_2_S as a preconditioning trigger molecule, by using different micromolar concentrations of an inorganic H_2_S donor, namely, sodium hydrosulfide (NaHS). Specifically, we investigated whether a 24-hour treatment could trigger a cascade that would enhance the endothelial response after an ischemic insult in an *in vitro* model of human microvascular endothelial cells (HMEC-1). The *in vitro* model is particularly feasible to address functional recovery of ECs since it allows exposure to hypoxia and drugs while maintaining the ability to proliferate and migrate and, therefore, performing experimental assays.

## 2. Materials and Methods

### 2.1. Cell Culture

Human microvascular endothelial cells 1 (HMEC-1) (ATCC® CRL3243™) were cultured in EndoGRO™-MV Complete Culture Media Kit (Millipore) and 1% penicillin/streptomycin (Pan Biotech) and passaged 80-90% confluence.

Dulbecco's Modified Eagle's Medium (DMEM) with phenol red (Sigma-Aldrich) 2% FBS (Microgem), 1% penicillin/streptomycin, and 1% L-glutamine (Microgem) was used for preconditioning and reoxygenation processes.

### 2.2. Hydrogen Sulfide Preconditioning and Ischemia/Reperfusion Protocol

Sodium hydrosulfide hydrate (NaHS) (Sigma-Aldrich) was used as a saline hydrogen sulfide donor. NaHS was freshly prepared in phenol red DMEM 2% FBS on the day of the experiment at a concentration of 5 mM; the stock solution was used for dilutions to reach our working conditions (1-10-100 *μ*M).

**Scheme 1:**
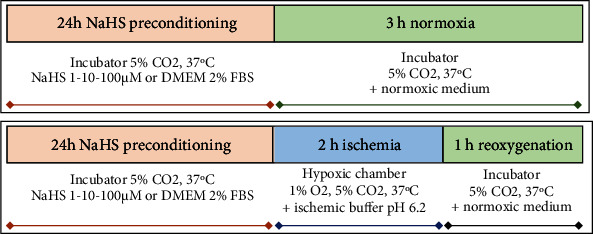
Schematic representation of the experimental design used. On the upper side is represented the “normoxia” setting, whereas on the lower panel is the setting used to simulate the *in vitro* “ischemia-reperfusion” condition.

*In vitro* simulation of ischemia/reperfusion injury was induced using an ischemic buffer (pH 6.2) as described in literature [25], and severe hypoxic conditions were reached through incubation in a sealed chamber (see Scheme 1) endowed with a regulator of gas mixture (InvivO_2_ 200, Ruskinn, United Kingdom).

A minimum level of 1% O_2_ can be achieved by this system, and no complete anoxia is guaranteed from the manufacturer. Moreover, to the best of our knowledge, 1% O_2_ at atmospheric pressure matches the oxygen partial pressure of ischemic tissues, including the myocardium [51]. The buffer was equilibrated overnight in a hypoxic chamber (1% O_2_, 5% CO_2_, 37°C). CO_2_ levels were set to 5% to buffer pH in medium, matching normoxic incubation. Cells were put in the hypoxic chamber, and the medium was changed with the equilibrated ischemic buffer. Ischemic protocol lasted for 2 hours in every experiment; cells were then reoxygenated with fresh medium for 1 hour in a normoxic incubator (37°C, 5% CO_2_).

### 2.3. Solutions and Reagents

#### 2.3.1. Ischemic Buffer

The buffer was freshly prepared before each experiment. The following formulation was used: NaCl 137 mM, KCl 12 mM, MgCl_2_ 0.49 mM, CaCl_2_ 0.9 mM, HEPES 4 mM, and Na L-lactate 20 mM (all purchased from Sigma-Aldrich). The ingredients were dissolved into double-distilled water, and pH was adjusted to reach 6.2 before bringing the solution to the required volume (100 mL).

#### 2.3.2. AZD6244

Also called Selumetinib, it is a selective MEK1/2 inhibitor. The inhibitor was solubilized in DMSO, as 10 mM stock solutions at -20°C. The stock was diluted to obtain a final concentration of 1 *μ*M.

#### 2.3.3. SCH772984 (Aurogene)

Selective ERK1/2 inhibitor was dissolved in DMSO, as 5 mM stock solutions at -80°C. The stock was diluted to reach a working concentration of 1 *μ*M.

#### 2.3.4. Antibodies

Primary antibodies anti-Heat-shock protein 90 (HSP90), p44/42 MAPK (ERK1/2), and phospho-p44/42 MAPK (p-ERK1/2) were all purchased from Cell Signaling Technology (The Netherlands). Secondary anti-rabbit antibody was purchased from ImmunoReagents, Inc. (North Carolina, USA). All antibodies were used according to the manufacturer's instructions.

### 2.4. Viability Assay

In order to address how cell viability could be influenced by preconditioning, an MTT assay was performed at 24 hours from the end of reoxygenation. Cells were plated in a 96-well plate at a density of 0.5 × 10^4^ cells/well in growth medium. On the next day, cells were preconditioned as previously described. After 24 hours of preconditioning, cells would undergo normoxic or IR condition following the ischemia/reperfusion protocol mentioned earlier. At the end of reperfusion, media was changed for all cells to DMEM 2% FBS, in order to keep cells alive for the next 24 hours but avoiding cell proliferation. After one day, MTT solution was added to each well (10 *μ*L/well), and plates were kept in the dark in an incubator for 3 hours. Then, media was removed and 100 *μ*L DMSO was added to each well, and absorbance was detected at 570 nm using a microplate reader. At least eight wells for each condition were analyzed.

### 2.5. Cell Migration Assay

To assess cell migration, cells were plated into three-chamber silicone-culture inserts (Ibidi) in a 12-well plate at a density of 4 × 10^5^ cells/mL in growth medium. This density was chosen as the most appropriate to have cells at 90-100% confluence overnight. On the next day, cells were treated with NaHS as described before, by removing the medium from each chamber and adding the desired treatment or just control medium, being careful not to scratch cells away. After 24 hours, all culture inserts were removed, and cells were gently washed with warm PBS with Ca/Mg. At this point, cells underwent IR protocol for 2 hours. At the end of the ischemia, cells were gently washed with warm PBS and growth medium was added to each well. After 6 hours of migration, cells were fixed in 4% PAF. Images were then acquired using a Nikon Eclipse Ti-E microscope with a ×10 lens. The MetaMorph software was used to both acquire and analyze all images. Cell motility was expressed as percentage of wound closure. At least three fields for each condition were analyzed.

### 2.6. In Vitro Angiogenesis Assay

The formation of capillary-like structures *in vitro* was studied on growth factor-reduced Matrigel (Corning, USA). Matrigel was used according to the manufacturer's instructions. ECs were plated into Petri dishes and treated according to our protocols on the following day. After *in vitro*-simulated IR injury, cells were seeded at 2.5 × 10^4^ cells per well onto Matrigel-precoated 24-well plate in growth medium. After 16 hours, cell organization was observed using a 5x lens. Images were acquired using the Infinity Analyze software (Lumenera Corporation). A minimum of three fields was analyzed for each condition.

Image analysis was performed using ImageJ's plugin Angiogenesis Analyzer, which works on phase contrast (RGB colors, 24 bit) or fluorescence images (8 or 16 bit). In order to avoid artifacts, some background noise had to be removed from the images using the “blurred mask tool.” Once the image has been modified, the program can be run and the images are automatically analyzed. The images are returned with different paths traced in a color-code mode and a table with different automatically measured parameters. We choose to include in our analysis three parameters: (I) number of master segments, (II) total master segment length, and (III) number of isolated segments.

### 2.7. Quantification of Mitochondrial Mass and Membrane Depolarization

To measure the depolarization of mitochondrial membrane in relationship with mitochondrial mass, endothelial cells were plated at a density of 2 × 10^4^ cells/well on 1.5 mm ø cover slips in growth medium. Cells were treated as previously described (see Hydrogen Sulfide Preconditioning and Ischemia/Reperfusion Protocol) with 1 *μ*M NaHS. After reperfusion, all specimens were incubated in DMEM 2% FBS with both MitoTracker™ Green FM (Thermo Fisher) 200 nM and MitoTracker™ Red CMXROS (Thermo Fisher) 50 nM in the dark for 30 minutes at 37°C. After staining, cells were washed with PBS and fixed with 4% PAF. After fixation, coverslips were mounted on microscope slides and observed at a confocal microscope (Zeiss LSM800) with a 63x oil-immersion objective. GMT has an absorption/emission spectrum of 490 nm/516 nm, while the RMT one is 579 nm/599 nm. Images were acquired with ZEN System software with a resolution of 512 × 512 pixels.

### 2.8. Western Blot

Cells were seeded into Petri dishes and were allowed to reach confluence before being treated with NaHS (1-10-100 *μ*M) and/or AZD6244 1 *μ*M and then underwent IR simulation protocol. At the end of the protocol, cells were scraped and lysed with RIPA buffer (Sigma-Aldrich), containing protease and phosphatase inhibitors (Complete protease inhibitor tablets and PhosStop, Roche). Whole cell lysates were separated by SDS-PAGE and wet-transferred on PVDF membranes, according to the manufacturer's protocol (Bio-Rad). After transfer, membranes were blocked in 4% nonfat dried milk or 4% BSA (depending on the primary antibody), for 1 hour at room temperature. Membranes were washed once in TBST and incubated with antibodies against HSP90 (1 : 1,000), ERK1/2 (1 : 1,000), and phospho-p44/42 MAPK (Erk1/2) (Thr202/Tyr204) (1 : 1,000) overnight at 4°C. Membranes were washed with TBST three times for 10 min. Anti-rabbit secondary antibody incubation (1 : 5,000) lasted for 1 hour. After another 30′ of washing with TBST 1x, chemiluminescence was detected using the ECL system (Bio-Rad) using a ChemiDoc Touch instrument (Bio-Rad).

### 2.9. Image Analysis

#### 2.9.1. Tubulogenesis Assay

During the analysis, we kept into consideration the following parameters: number of master segments, number of master junctions, total master segment length, and number of isolated segments. A well-constructed network should have more master elements (segments and junctions), thus creating a more physiological and functional structure. The images were analyzed with ImageJ's Angiogenesis Analyzer plugin, and only the aforementioned parameters were kept into consideration. All images were converted to RGB format if needed.

#### 2.9.2. Mitochondrial Staining

Multichannel images were split to separate the two channels. Then, the corrected total cell fluorescence (CTCF) was calculated for each cell in both channels as described in literature [52]. For each cell, the ratio between mitochondrial depolarization and mass was calculated and normalized on the control condition.

#### 2.9.3. Image Lab Quantification

Using Image Lab software, bands were detected automatically and then subtracting the background to obtain a better quantification. Density of bands was computed keeping into consideration the value of the integrated density adjusted by subtracting the background. All proteins were quantified by normalizing the density over the loading control (HSP90).

### 2.10. Statistical and Computer Analysis

Results are expressed as mean ± SEM. Differences between groups were analyzed by one-way ANOVA or Kruskal-Wallis or Mann–Whitney *t*-test. Significance was established at *p* value < 0.05. All raw data were analyzed using Microsoft Excel and Prism 6. All experiments were repeated at least three times.

## 3. Results

### 3.1. Biological Effects of NaHS Preconditioning

During acute myocardial infarction, endothelial cells can go towards death through apoptosis, necrosis, or autophagy [53–55]. To assess endothelial cell response after IR injury in terms of viability, an MTT assay was performed after 24 hours from the injury.

**Figure 1:**
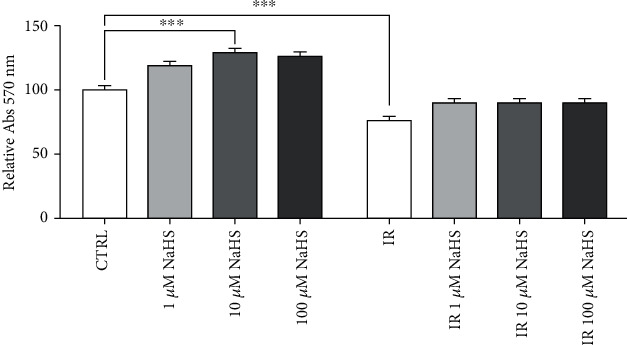
Normalized viability on the normoxic control condition. Statistical significance was set at *p* value < 0.05 (^∗∗∗^*p* ≤ 0.001).

As shown in Figure 1, there was a decrease in the IR condition compared to normoxia, but H_2_S did not significantly increase cell viability in the post-IR condition.

The highest viability was observed at 10 *μ*M, but only in normoxic conditions.

**Figure 2:**
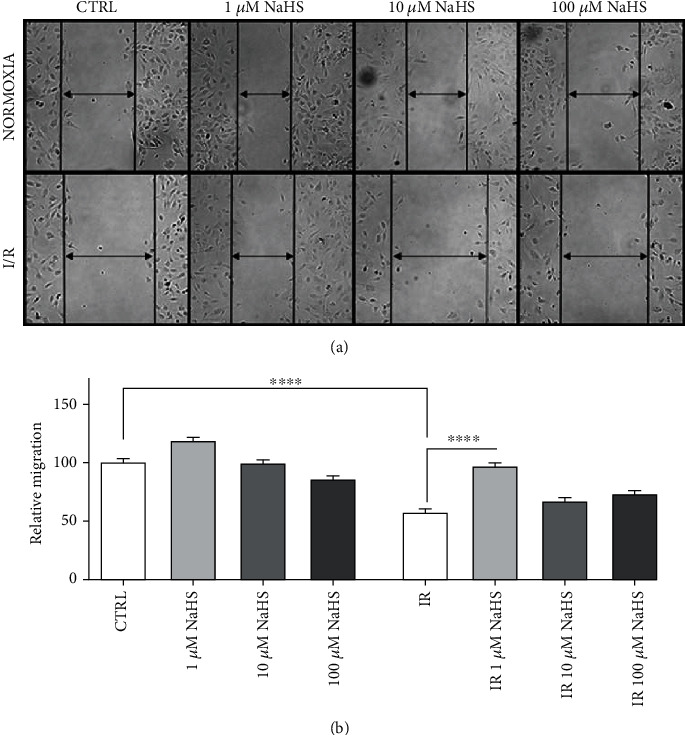
(a) Representative images of cells after 6 hours in the different experimental conditions. (b) Relative migration, all data sets were normalized on the normoxic control condition (CTRL). Statistical significance: ^∗∗∗∗^*p* < 0.0001.

Cell migration is one of the essential processes during angiogenesis. In fact, cells have to migrate to create new vessels. The creation of new vessels is important after IR injury in order to allow an adequate blood supply to the myocardium and, eventually, to replace arteries that have been disrupted by the ischemic event and subsequent reperfusion. To study this process, we observed cell migration following IR simulation. After 6 hours, the percentage of wound closure was calculated and compared to the normoxic control condition (Figure 2(b)).

We observed a significant improvement of cell migration in cells treated with NaHS, with the strongest effect at 1 *μ*M NaHS, both in normoxia and post-IR.

To study whether NaHS treatment had a proangiogenic effect on cells that underwent IR injury simulation, we studied their ability to form capillary-like structures *in vitro* focusing on the most promising concentration of 1 *μ*M NaHS.

**Figure 3:**
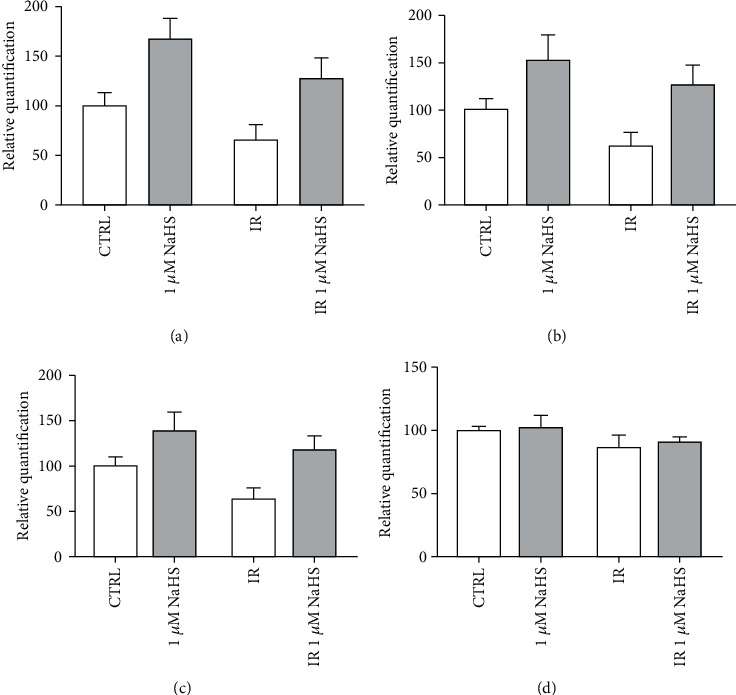
Quantification of main elements of the *in vitro* capillary network: (a) total master segment length; (b) number of master segments; (c) number of junctions; (d) number of isolated elements.

After IR, cells were detached and plated in Matrigel-precoated 24-well multiwell in EndoGRO 10% FBS for 16 hours. As shown in Figure 3, there is a positive trend in terms of capillary formation in both normoxia and IR, upon treatment of cells with 1 *μ*M NaHS, but no statistical significance was observed.

We decided to perform a double MitoTracker™ staining on all samples to investigate whether mitochondria were affected by NaHS preconditioning. MitoTracker™ Green FM stains all mitochondria, whereas MitoTracker™ Red CMXROS stains only viable mitochondria. Red fluorescence is directly proportional to membrane depolarization.

By calculating the ratio between red and green fluorescence, we determined the influence of NaHS and IR on mitochondria in endothelial cells.

**Figure 4:**
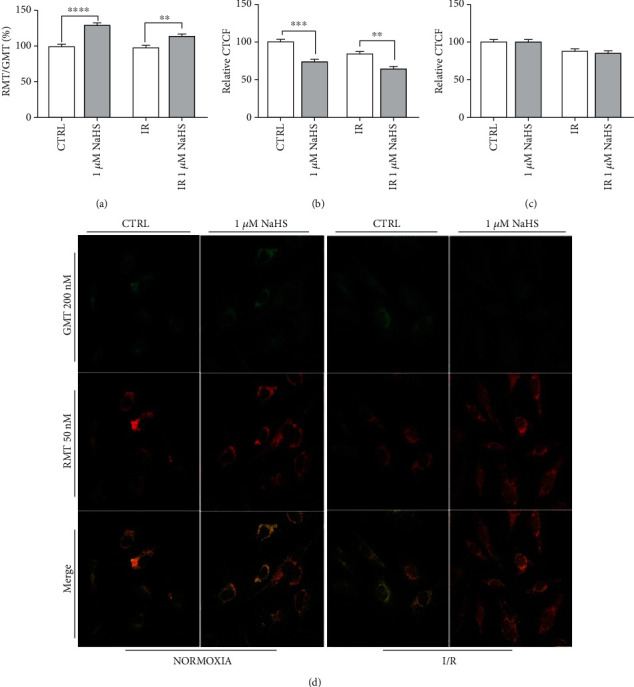
(a) Ratio between red (RMT) and green (GMT) MitoTracker™; (b, c) GMT and RMT fluorescence, respectively, calculated for each experimental condition as a percentage of CTRL; (d) representative fields of stained cells. All data sets were normalized on the normoxic control condition (CTRL). Statistical significance: ^∗∗^*p* < 0.01, ^∗∗∗^*p* < 0.001, and ^∗∗∗∗^*p* < 0.0001.

NaHS treatment decreased the mitochondrial mass with a similar fashion in normoxia and IR (Figure 4(b)). However, the mitochondrial function did not change much between the two IR experimental conditions. On the other hand, NaHS treatment caused an improvement in mitochondria viability, observed as an increased ratio between vital (red) and total (green) mitochondria, both in IR and normoxic conditions (Figures 4(a)–4(d)).

### 3.2. NaHS Preconditioning and IR Injury Influence ERK1/2 Phosphorylation

Since ERK1/2 is deeply implicated in both cell migration and angiogenesis, but also in inducing apoptosis, we evaluated whether IR and/or NaHS treatment could influence its level of phosphorylation. Protein expression and phosphorylation levels were assessed through western blot.

**Figure 5:**
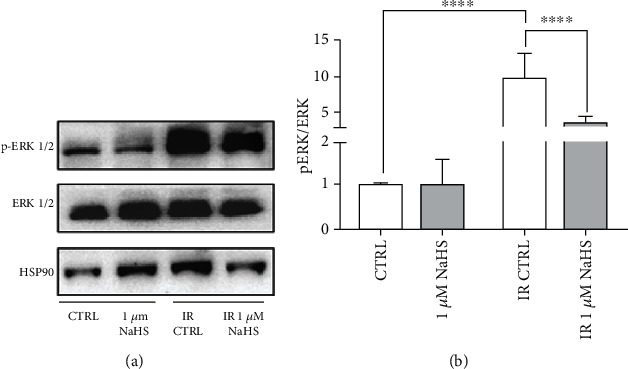
(a) Representative experiment of ERK1/2 and p-ERK1/2; (b) ratio of p-ERK on total ERK (*n* = 4). Both total and phosphorylated proteins were first normalized on the loading control (HSP90), and then, the ratio was calculated. All data sets were normalized on the normoxic control condition (CTRL). Statistical significance: ^∗∗∗∗^*p* < 0.0001.

ERK appeared to be strongly phosphorylated in IR condition, whereas NaHS treatment attenuated the p-ERK/ERK ratio, bringing it towards a more physiological condition (Figure 5).

### 3.3. Effects of MEK-ERK Pathway Inhibition

After assessing the levels of ERK phosphorylation, we decided to investigate if NaHS directly modulated the ERK pathway.

At first, we targeted its upstream modulator, MEK, using its specific inhibitor AZD6244 before preconditioning. Both viability and cell migration were tested.

Considering cell viability, there was no relevant difference with or without inhibitor and/or 1 *μ*M NaHS, confirming what we had already observed (see Figure 1).

**Figure 6:**
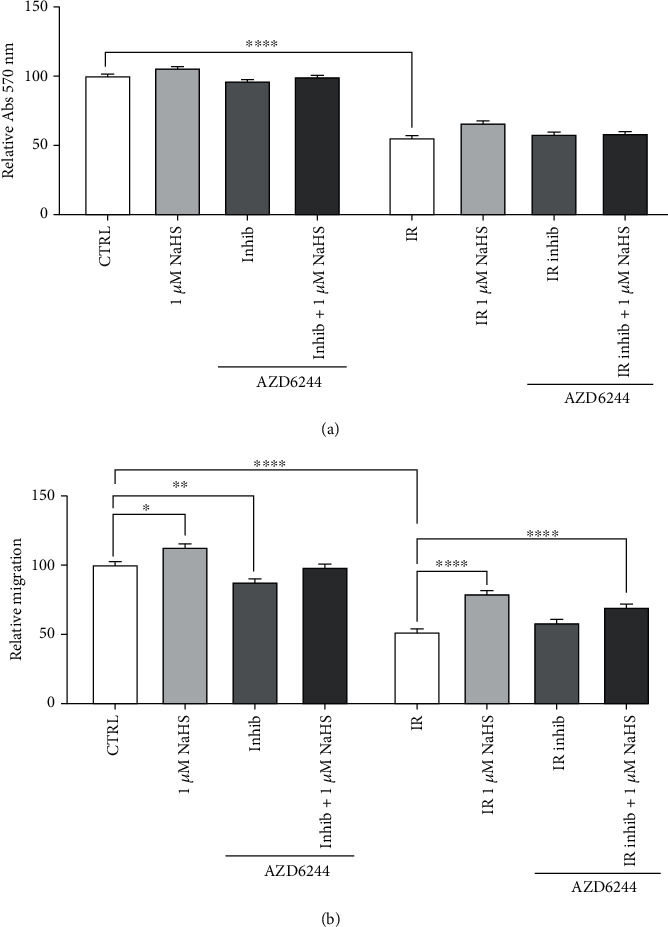
(a) Cell viability after MEK inhibition (1 *μ*M AZD6244) and 1 *μ*M NaHS preconditioning. (b) Cell migration after MEK inhibition (1 *μ*M AZD6244) and 1 *μ*M NaHS preconditioning. Statistical significance: ^∗^*p* < 0.05, ^∗∗^*p* < 0.01, and ^∗∗∗∗^*p* < 0.0001.

When we tested the effects on cell migration, we confirmed the effect of preconditioning with 1 *μ*M NaHS in both normoxia and IR. Moreover, we observed that the treatment with MEK inhibitor only slightly decreased the rate of cell migration compared to the control condition, especially in normoxia. An even slighter effect was observed when we used both the inhibitor and NaHS (Figure 6(b)).

After evaluating the effects on upstream inhibition, we decided to investigate whether NaHS preconditioning could rescue the biological effects after direct ERK1/2 inhibition.

Again, we assessed the inhibition on both cell viability and cell migration, which are two key pathways in which ERK1/2 is strongly involved.

**Figure 7:**
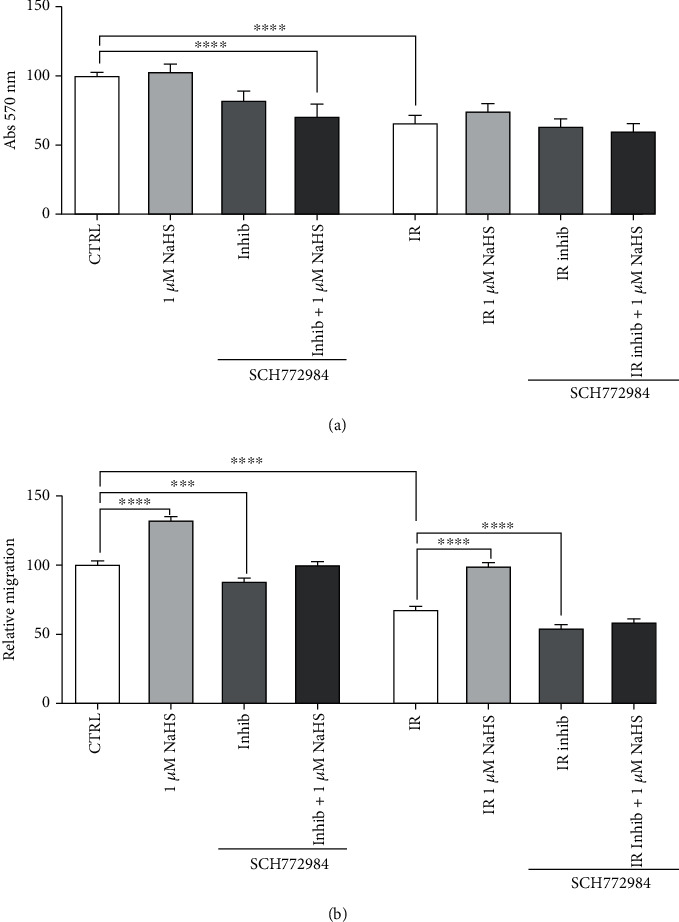
(a) Normalized viability after direct ERK1/2 inhibition (1 *μ*M SCH772984) and 1 *μ*M NaHS preconditioning. (b) Cell migration on 1 *μ*M NaHS preconditioned cells after ERK1/2 inhibition (1 *μ*M SCH772984). Statistical significance: ^∗∗^*p* < 0.01, ^∗∗∗^*p* < 0.001, and ^∗∗∗∗^*p* < 0.0001.

No relevant changes were observed in IR condition, but a significant decrease in cell viability was observed in normoxia when cells were treated with both ERK inhibitor and NaHS (Figure 7(a)).

Focusing on cell migration, in normoxia, ERK inhibition caused a significant decrease in cell migration, but NaHS treatment was able to rescue this inhibition, restoring the migration (Figure 7(b)). However, in IR, the direct inhibition of ERK actually caused a very strong decrease in cell migration when compared to the IR untreated cells, which could not be rescued by NaHS treatment (Figure 7(b)).

## 4. Discussion

Cardiovascular diseases are the leading cause of death worldwide. In 2016, they represented up to 30% of all global deaths [56, 57]. CVDs include different clinical conditions, most of which are associated to a pathological condition known as ischemia/reperfusion injury, such as myocardial infarction and stroke [58–61]. Whether this phenomenon affects the clinical outcome, it depends on the site, the duration, and the severity of the ischemic event itself. Nevertheless, postischemic reperfusion could exacerbate the damage both locally and systemically. This paradox relies on an increase in inflammatory response and, later on, to a profound tissue injury [2]. During an ischemic insult, moreover, there are several biochemical and cellular changes caused by the rapid depletion of oxygen and nutrients that cells cannot bear altogether with exacerbated effects during the reperfusion phase [62–64].

Endothelial integrity is pivotal for a proper vascularization which is, in turn, a key step for restoration of the physiological state as demonstrated by the several complications for patients undergoing incomplete cardiac revascularization [65, 66]. In this regard, it is reported that endothelial cells are very sensitive to IRI, even more than other cell types [20, 22, 27]. In addition, they are critical mediators for the onset of the inflammatory response, the generation of ROS, and the rapid restoration of the physiological pH.

In the ischemic myocardium, these events negatively affect the surrounding tissue, exacerbating the reperfusion injury and worsening the outcomes [2, 67].

A considerable part of literature about IRI has its focus on cardiac cells, while a small amount of data is available about endothelial cells, despite their importance in this context. Considering the role of ECs in both injury and recovery after the ischemic insult, the role of endothelial function in the hypoxic/ischemic *scenario* is of scientific relevance to ameliorate tissue recovery.

To this end, the action of hydrogen sulfide as a protective agent on endothelial cells against hypoxic/ischemic stress is promising because of the recent description of its beneficial effects in similar settings [68, 69]. H_2_S is known to act as a secondary messenger [70, 71], and there is evidence that it can be beneficial in IRI in several experimental models [72, 73]; hence, its potential as endogenous and exogenous therapeutic agent is gaining scientific attention. In order to study the effects solely on the endothelium, we decided to use an *in vitro* model of human microvascular endothelial cells to test the direct effects of hydrogen sulfide as a preconditioning agent, by using the inorganic donor NaHS. The aim was to recover the physiological state after the ischemic event. In particular, we expected to trigger a response in endothelial cells that would last until the end of the ischemic event, although NaHS is known to be rapidly hydrolyzed in water, establishing equilibrium between H_2_S and S^2−^+HS^−^ species [74].

The preconditioning phenomenon can play an important role in the attenuation of ischemic consequences as demonstrated by the modulation of angiogenic activity of ECs. We verified this hypothesis by addressing the effects of H_2_S on cell migration and the ability to create capillary-like structures *in vitro*. Data showed that 1 *μ*M NaHS pretreatment in cells that underwent IR protocol was able to rescue the defect in the migration rate, restoring the properties observed in the control-normoxic condition. This finding suggests a potential role of the gasotransmitter in promoting tissue repair following IRI.

There is still open debate about the actual *in vivo* levels of endogenous hydrogen sulfide. In order to test different conditions, the comparison of 2 putative concentrations (1 and 10 *μ*M) can help decipherer the biological effects of constitutive deficiency. Low levels, 1 *μ*M NaHS, restored the physiological condition in cell migration, whereas 10 *μ*M gave a less intense increase in consistence with previously described bimodal activities shown in other cell types [44]. These data contribute to the hypothesis that endogenous and exogenous H_2_S, and their respective levels, might have opposite effects on some important cellular functions.

Goubern et al. suggest that exogenous H_2_S could function as an electron donor and as a potential inorganic energy source in mammalian cells at low micromolar concentrations [75]. The described mitoprotectant behavior could involve mitochondrial biogenesis and dynamics, as demonstrated in an endothelial model of rat aortic endothelial cells (RAEC) in which exogenous H_2_S exerts antifission and promitophagy effects when compared to the control in which endogenous H_2_S is modulated [76]. An enhanced mitophagy could explain diminished mitochondrial content in preconditioned I/R cells compared to the untreated control (Figure 4).

Despite the decrease in the mitochondrial mass, the ratio of functional mitochondria was enhanced in cells pretreated with NaHS. These data could be explained by an H_2_S-mediated enhancement of mitochondrial function and its suggested role of ROS scavenger.

The next step was to understand whether a 24-hour preconditioning could trigger a modulation of ERK phosphorylation, because of the role that the MEK/ERK pathway has in endothelial cell migration, other than apoptosis. Most interestingly, IR alone significantly enhanced ERK1/2 phosphorylation, while 1 *μ*M NaHS treatment managed to drastically reduce this effect, going towards a more physiological (CTRL) phosphorylation state. On the other hand, the treatment did not affect ERK1/2 phosphorylation in normoxic cells.

Last, since it appeared that ERK was modulated by the NaHS treatment, we inhibited both ERK1/2 and its upstream activator, MEK, with the selective inhibitors AZD6244 and SCH772984. After testing the effects on cell viability and cell migration, we observed that the direct inhibition of ERK, but not MEK, decreased cell migration and could not be rescued by H_2_S treatment, thus indicating that H_2_S modulates the ERK pathway acting at the level of ERK phosphorylation.

Further experiments are needed to better describe the involvement of this pathway activated 24 hours ahead of the ischemic insult and how it is sustained over time. Moreover, it might be interesting to see whether the same concentrations could trigger similar effects in the postconditioning window and what similarities the pre- and postconditioning *scenarios* might have in common.

## 5. Conclusion

In our study, we showed that, despite its rapid metabolization, H_2_S could be used as a long-term preconditioning agent to ameliorate the effects of IRI. Moreover, this work is aimed at investigating the role of H_2_S as a modulator of an ERK1/2-dependent pathway, thus suggesting an important role for microvascular endothelial cells to participate in the response to ischemia/reperfusion injury. A deeper comprehension of endothelial cells in this context can contribute to design better strategies to preserve and repair tissues after a hypoxic/ischemic insult.

## Data Availability

All the data used to support the findings of this study are included within the article.
